# Deprescribing in older patients with hyperpolypharmacy: a cluster-randomised trial in primary care

**DOI:** 10.1093/ageing/afag209

**Published:** 2026-07-19

**Authors:** Gert Baas, Mette Heringa, Sanne Verdoorn, Henk-Frans Kwint, Eman Badawy, Jacobijn Gussekloo, Jako Burgers, Petra Denig, Marcel L Bouvy

**Affiliations:** SIR Institute for Pharmacy Practice and Policy, Leiden, ZH, The Netherlands; Department of Pharmaceutical Sciences, Utrecht University, Utrecht 125, The Netherlands; SIR Institute for Pharmacy Practice and Policy, Leiden, ZH, The Netherlands; SIR Institute for Pharmacy Practice and Policy, Leiden, ZH, The Netherlands; SIR Institute for Pharmacy Practice and Policy, Leiden, ZH, The Netherlands; SIR Institute for Pharmacy Practice and Policy, Leiden, ZH, The Netherlands; Public Health and Primary Care, Leiden University Medical Center, Leiden, The Netherlands; Department of Family Medicine, Maastricht University, Maastricht, LI, The Netherlands; Dutch College of General Practitioners (NHG), Utrecht, The Netherlands; Clinical Pharmacy & Pharmacology, University of Groningen, University Medical Center Groningen, PO Box 30001 EB71, Groningen 9700RB, The Netherlands; Department of Pharmaceutical Sciences, Utrecht University, Utrecht 125, The Netherlands

**Keywords:** deprescribing, (hyper)polypharmacy, older people, medication review, randomised controlled trial

## Abstract

**Background:**

Polypharmacy increases the risk of adverse drug events and potentially preventable hospital admissions. Deprescribing may reduce medication-related harm, and a clinical medication review (CMR) provides an opportunity to support this.

**Objective:**

To investigate the effect of a deprescribing-focused CMR by trained pharmacists on the number of reduced and stopped medications among patients aged ≥75 years with hyperpolypharmacy using multidose-drug-dispensing (MDD)-systems.

**Methods:**

In this cluster-randomised controlled trial, pharmacists were trained to perform a deprescribing-focused CMR, while control pharmacies provided usual care. Eligible patients were aged ≥75 years, had hyperpolypharmacy and used MDD. Dispensing data were used to determine the number of reduced and stopped medications per patient after 6 months (primary outcome). Secondary outcomes were: total number of medications, health problems and quality-of-life (EQ-5D-5L, EQ-VAS). A generalised linear mixed model was used for the primary outcome.

**Results:**

A total of 318 patients from 58 pharmacies were enrolled (155 intervention, 163 control). At 6 months, patients in the intervention group had significantly more medications deprescribed than those in the control group (mean 2.5 vs. 1.7 per patient; mean difference 0.79, 95% CI 0.29–1.28; *P* = .002). There was no significant between-group difference in the total number of medications at 6 months (intervention −0.30 vs. control +0.12; *P* = .108). No significant differences were observed in health problems or quality-of-life.

**Conclusion:**

Deprescribing-focused CMRs by trained pharmacists successfully increased reduction and stopping of medications in older patients with hyperpolypharmacy using MDD-systems, while no changes in health problems or health-related quality of life could be observed over 6 months.

## Key Points

- A pharmacist-led deprescribing intervention increased medication discontinuation and reduction in older people.
- A deprescribing toolbox and training supports medication reviews to reduce and stop medication in older people.
- Despite deprescribing, total medication counts did not differ between intervention and control groups after 6 months.

## Introduction

More than half of older people live with multiple chronic conditions, leading to long-term use of multiple medications, often defined as polypharmacy (≥5 medications) or hyperpolypharmacy (≥10 medications) [1, 2]. While medications can be appropriate by improving health and preventing complications, polypharmacy is associated with increased hospital admissions, morbidity and mortality [3–5]. The risk arises from age-related physiological changes, comorbidities and complex medication regimens [6], and is exacerbated by inappropriate prescribing. In primary care, potentially inappropriate prescribing among older patients was identified in about one-third of patients [7–9]. In the Netherlands, one in five patients aged 75 years and older use multidose drug dispensing (MDD) systems [10] to support medication adherence, particularly among those with complex regimens and limited capacity for self-management [11]. MDD users represent a vulnerable group, as they generally use more medicines leading to hyperpolypharmacy [12, 13] and have a relatively high number of drug-related problems (DRPs) [9, 14]. Studies have also reported a poorer quality of drug treatment [12, 15–17] and fewer changes in ongoing therapy among MDD users compared with patients receiving their medicines through standard dispensing, suggesting a reduced reconsideration of treatment in this population [13]. Suboptimal prescribing and overtreatment remain concerns, highlighting the need for regular and collaborative medication review in this population [15, 16].

Given these risks, strategies to safely reduce overall medication use are needed for groups at highest risk. Deprescribing is the planned, health care provider (HCP)-supervised dose reduction or stopping of a medication [18]. Over the past decade, deprescribing has gained substantial international attention as a promising approach to address the risks of polypharmacy. Evidence shows that deprescribing is generally safe and can reduce medication use: a review of randomised trials on deprescribing interventions in older patients with polypharmacy reported that almost all interventions (13/14) reduced medication counts without adverse effects, with several also leading to improvements in health-related quality of life, reduced health care costs or fewer hospitalisations [19]. Furthermore, patient-centred deprescribing interventions achieve persistent reductions in drug use, with most discontinued medications remaining stopped after 12 months [20]. Among older people with frailty, studies suggest that deprescribing is safe and feasible, with reductions in the number of medications and potentially inappropriate medications [21]. So far, no studies have assessed the impact of deprescribing initiatives among older patients with hyperpolypharmacy using MDD systems.

Deprescribing can be integrated into a CMR, a well-established intervention in primary care that brings together general practitioners (GP), community pharmacists (CP) and patients. Its design inherently supports deprescribing: prescribers and pharmacists jointly assess the appropriateness of current pharmacotherapies, beginning with a patient consultation to identify current health status, goals and preferences. Previous trials in the Netherlands demonstrated that CMRs can improve patient-reported outcomes in older patients with polypharmacy [22]. However, they did not explicitly focus on deprescribing, leaving it unclear whether a more targeted approach could be more effective. To explore this, we conducted a mixed-methods feasibility study of a deprescribing-focused CMR among older MDD users with hyperpolypharmacy [23]. The study showed that the intervention was acceptable to both patients and HCPs, and that deprescribing recommendations were implemented in most patients.

This study aimed to determine whether a deprescribing-focused CMR by trained pharmacists increases the number of reduced and stopped medications after 6 months, compared with usual care in older patients with hyperpolypharmacy using MDD systems.

## Methods

### Study setting and design

A pragmatic cluster-randomised controlled trial was conducted in 58 community pharmacies in the Netherlands comparing a CMR focused on deprescribing with usual care. Pharmacies were recruited between March 2022 and November 2022 via mailings and information letters distributed through an academic pharmacy network [24]. Before patient recruitment started, pharmacies were randomised in 2 arms (29 intervention, 29 control) by an independent researcher Adrianne Faber (AF), who was not otherwise involved in study conduct, using block randomisation with a block size of four to obtain equal numbers of pharmacies per group. Pharmacies within the same pharmacotherapy audit meeting (PTAM) local network were placed in the same block to avoid contamination. Because a PTAM network in the Netherlands rarely exceeds four pharmacies, the maximum block size was set at four. Due to the nature of the intervention, blinding of HCPs and patients was not possible. The random allocation sequence was generated and held exclusively by the AF and was not accessible to recruiting personnel. Formal blinding of outcome assessors was also not applied. CPs in the intervention group received a one-day face-to-face training. CPs in the control group only attended a 1-hour online session solely focused on explaining study procedures and patient recruitment, ensuring consistency across study groups. Available pharmacy and pharmacist characteristics are presented in Supplementary Appendix I. Patient and public involvement informed the development of the intervention and study materials. Representatives from patient advocacy organisations (Zorgbelang Inclusief) and an older persons’ advisory panel (Ouderenberaad ZHN) provided feedback on study procedures and patient-facing materials, which were refined accordingly.

### Study participants

Eligible patients were community-dwelling adults aged ≥75 years using an MDD system and ≥10 chronic medications (hyperpolypharmacy). Lists of potentially eligible patients were generated by CPs from their pharmacy information system and screened for exclusion criteria in collaboration with GPs. Patients were excluded by either CP or GP, using routinely documented information in the medical records, if they had: (i) an estimated life expectancy of ≤6 months, (ii) cognitive impairment (as the intervention relied on patient participation in consultations and shared deprescribing decision-making), (iii) residence in nursing homes, (iv) CMR within the past 12 months and (v) repeat prescriptions issued exclusively by a hospital specialist. Patients with a CMR within the past 12 months were excluded to avoid overlap with recently implemented medication optimisation interventions that could influence baseline medication use and reduce the ability to detect effects attributable to the study intervention.

Following pharmacy randomisation, eligible patients were recruited between October 2022 and March 2024. Patients were approached by telephone by CPs who had been instructed to contact patients without applying any fixed order (e.g. alphabetical or first-listed) or reviewing additional patient information to select patients. Those expressing interest received an information package, including an informed consent form, which they returned directly to the research team. Upon receipt, the researchers informed the relevant CPs of the patient’s inclusion. Participating patients were followed for 6 months.

### Multilevel intervention

The intervention targeted both HCPs and patient level and consisted of complementary implementation and care components.

#### Deprescribing training and implementation support (HCP level)

The one-day face-to-face training for the intervention group was developed based on previous training programs [25] and the Dutch deprescribing guideline module to support deprescribing-focused CMRs [26]. The training focused on: (i) the organisation of CMR focusing on deprescribing; (ii) communication skills to support deprescribing consultations; (iii) the application of recently developed drug-specific deprescribing factsheets in practice [26]. The training combined educational methods including interactive plenary teaching, case-based learning, role-play, peer discussion and feedback using practical polypharmacy cases relevant to older adults. During the training, the CPs received a toolbox with the following materials: (i) ten drug-specific deprescribing fact sheets; (ii) an information letter for GPs and (iii) a PowerPoint presentation to be used in a local PTAM, covering the same aspects as in the CPs’ training and (iv) consultation aid (A4) with tips and tricks for deprescribing consultations.

#### Deprescribing-focused clinical medication review (patient level)

**Figure 1:**
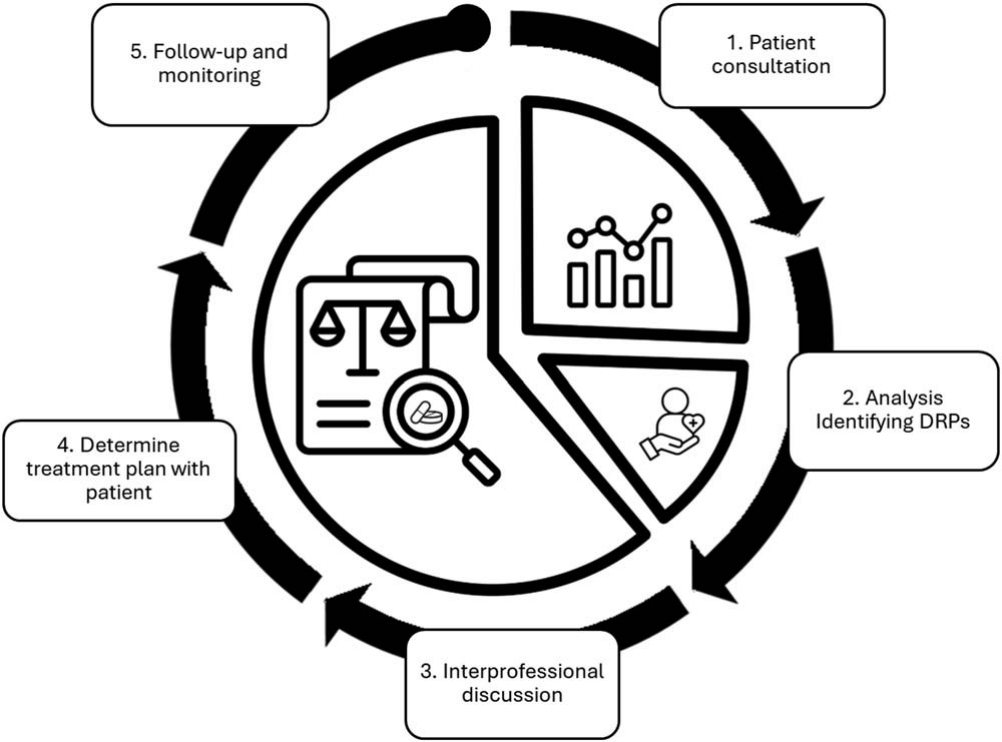
Five step approach of a CMR according to the Dutch multidisciplinary guideline ‘Polypharmacy in the Elderly’ [27] with special focus on deprescribing. The CMR begins with a consultation (step 1) between the patient and the CP, using input from the questionnaires to understand the patient’s preferences and health-related issues. This includes identifying any medications the patient prefers to continue or discontinue and any adverse effects they may be experiencing. This is followed by a pharmacotherapeutic analysis by the CP to identify potential DRPs and opportunities for deprescribing (step 2). The CP and GP then discuss the identified DRPs, patient preferences and possible actions, including deprescribing (step 3). If needed, the CP or GP consults other HCPs. Agreed deprescribing proposals were subsequently discussed with the patient and incorporated into a pharmaceutical care plan (step 4). Implementation took place through routine procedures. All deprescribing actions will be monitored through follow-up (step 5), which includes patient interaction by either the CP, nurse practitioners or GPs, and with interprofessional communication as needed, to adjust tapering based on the patient’s health or withdrawal symptoms.

The deprescribing-focused CMR was based on the Dutch multidisciplinary guideline on polypharmacy in older people (2019) and the deprescribing module (2020) as part of the guideline [26, 27]. The CMR followed a five-step process (Figure 1): (i) patient interview identifying current health problems, goals and preferences, (ii) pharmacotherapeutic analysis using the guideline module, (iii) discussion and consensus with the GP, (iv) discussion and consensus with the patient about a pharmaceutical care plan and implementation of actions and (v) follow-up monitoring of actions and effects through patient interaction. Before the CMR took place, patients completed questionnaires on their health-related complaints and preferences.

### Usual care

Patients in the control group received usual care, and no training or deprescribing toolbox was provided to HCPs. Within the framework of usual care, HCPs retained the discretion to perform a CMR whenever deemed appropriate.

### Outcome measures and data collection

The primary outcome was the number of medications stopped or reduced per patient at 6 months. Dispensing data over a 24-month period, covering a maximum of 18 months before and at least 6 months after inclusion for all study patients, were provided by Dutch Foundation for Pharmaceutical Statistics (Stichting Farmaceutische Kengetallen: SFK). SFK maintains a comprehensive national database of dispensing data provided by over 98% of community pharmacies and reflecting medication exposure in a population of approximately 16.5 million individuals (www.sfk.nl). For the analysis, deprescribing was defined as stopping a medication, reducing its dose or substituting it for an alternative with lower potency or reduced complexity, observed after 6 months using a stepwise approach applied for all medication classes. Dose reductions and substitutions to less potent or less complex alternatives were both categorised as medication reduction. At the ATC-5 level, medications were checked for continuation. If present after 6 months, the daily dose was assessed to identify dose reductions. If not present, substitutions within the same therapeutic group (ATC-2 level) were identified. In such cases, equivalence and potency were evaluated according to the deprescribing factsheet [26] (or, when absent, primary care treatment guidelines), documenting lower potency as ‘reduced’. In the absence of guidelines, changes in medication complexity (e.g. number of active substances, number of devices and dosing schedules) were assessed to determine whether the substitution represented ‘reduced’, ‘similar’ or ‘increased’ medication use. If no substitution was identified, the medication was documented as ‘stopped’. Two researchers (E.B. and G.B.) independently reviewed dispensing records to identify deprescribing using predefined criteria applied on dispensing data obtained from the national SFK database. The criteria were based on the Dutch deprescribing fact sheets and guidelines from the Dutch College of General Practitioners (Nederlands Huisartsen Genootschap: NHG).

Secondary outcomes included the total number of medications in use, the number and type of health problems with an impact on daily life [22, 28], health-related quality of life measured with the EQ-5D-5L and the EQ Visual Analogue Scale (VAS), the number and type of DRPs identified during CMR, and the implementation rate of deprescribing-related recommendations. Health problems and health-related quality of life were assessed through patient questionnaires at baseline and 6 months. Demographics, the Integrated Systematic Care for Older People (ISCOPE) questionnaire [29] and Self-Perceived Burden Scale Questionnaire questionnaire [30] were completed at baseline to evaluate the presence of complex problems, and inadequate health literacy. Questionnaires were completed by telephone interviews, on paper or via a digital link sent by email, depending on patient’s preference. All responses were entered into CASTOR Electronic Data Capture (CASTOR EDC) by the researchers.

CMR data were documented by CPs in CASTOR using Hepler and Strand’s classification of DRPs [31], including proposed actions and their implementation status. Two researchers (E.B. and G.B.) independently checked CMR data for completeness and consistency, resolving disagreements with CPs and/or a third researcher.

### Sample size

The sample size calculation was based on detecting a difference of one deprescribed medication per patient after 6 months, assuming a standard deviation of 2.5, a two-sided α of 0.05, power of 80%, an intra-cluster correlation coefficient (ICC) of 0.05 and an average cluster size of seven patients. This required 38 clusters (266 patients). To allow for an expected 30% patient dropout, the target was increased to 10 patients per pharmacy. After adjusting for a 20% potential pharmacy dropout, the total target was 46 pharmacies.

### Statistical analyses

Baseline characteristics were compared between the intervention and control group. Categorical variables were analysed using Pearson’s chi-square test. Continuous variables were assessed for normality using visual inspection of histograms and the Shapiro–Wilk test; as all continuous baseline variables were non-normally distributed, between-group differences were evaluated using the Mann–Whitney U test, and results are presented as medians with interquartile ranges (IQR).

Analyses were based on intention-to-treat, including all randomised patients with at least 4 months of medication dispensing data after baseline; patients with less dispensing data were excluded. A per-protocol analysis was additionally performed, excluding patients who, based on dispensing data verification, were found not to meet the predefined inclusion criterion for hyperpolypharmacy (≥10 chronic medications at baseline) and, within the intervention group, those who did not receive the intervention. The primary outcome (the number of medications stopped or reduced per patient at 6 months) was estimated using a Generalised Linear Mixed Model (GLMM) with a linear distribution, including intervention group as a fixed effect and a random intercept for cluster (pharmacy) to account for intra-cluster correlation. No covariates were included in the primary model. To assess whether the intervention effect differed across patient characteristics, we evaluated effect modification by sequentially adding prespecified covariates (sex, age, baseline medication count and ISCOPE complexity score) and their interaction terms with the intervention to the GLMM. Residuals at both patient and cluster level were assessed for normality using the Shapiro–Wilk test and visual inspection of quantile–quantile (QQ) plots and histograms.

Secondary outcomes were analysed using GLMMs. For questionnaire-based outcomes assessed at baseline and 6 months (number of health problems, EQ-5D-5L and EQ-VAS), models included fixed effects for intervention, time and their interaction, with baseline values as covariates, following an analysis of covariance (ANCOVA) approach. Time (in months) was included to adjust for potential temporal trends over the 6-month follow-up. Outcomes derived from the CMR process or dispensing data (DRPs, implementation of deprescribing-related recommendations and medication changes) were analysed using GLMMs including intervention as a fixed effect and cluster as a random effect, without inclusion of time.

When a statistically significant interaction with intervention was identified, pairwise contrasts were performed to estimate the intervention effect to obtain the corresponding *P*-values.

All statistical analyses were performed in IBM SPSS Statistics version 29.0.1.0 (171) (IBM Corporation, Armonk, NY, USA).

### Ethics and confidentiality

The Medical Research Ethics Committee NedMec determined that the study (21/672) was not subject to the Medical Research Involving Human Subjects Acts. The study protocol UPF2211 was approved by the Institutional Review Board of UPPER. All patients gave written informed consent. To ensure patient privacy, all data were anonymised. Data collection was conducted using CASTOR, a secure online data management platform. The trial was registered at ClinicalTrials.gov (ID: NCT05609981), where the study protocol and statistical analysis plan can be accessed. A completed CONSORT extension checklist for cluster randomised trials is provided in Supplementary Appendix II.

## Results

**Figure 2:**
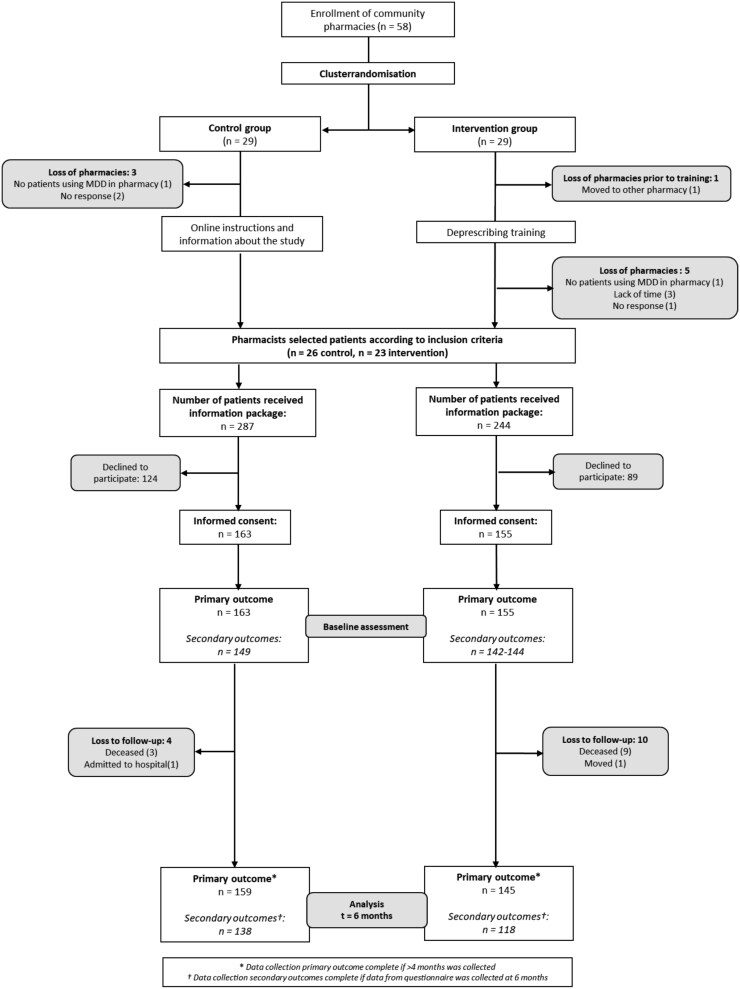
Flow chart study population.

A total of 58 community pharmacies were enrolled in the study, with 29 randomised to the intervention group and 29 to the control group (Figure 2). In the intervention arm, 28 CPs completed the one-day deprescribing training, while 26 CPs in the control arm received online instructions.

After training the participating CPs, 244 patients in the intervention group and 287 patients in the control group received an information package by post after being contacted by telephone by their CP. Of these, 89 patients in the intervention group and 123 in the control group declined participation. This resulted in 155 patients in the intervention group and 163 in the control group providing written informed consent (range per pharmacy: 1–18). Patient baseline characteristics for both groups are presented in Table 1. Over the 6-month follow-up, ten patients in the intervention group (nine deceased, one moved) and four in the control group (three deceased, one hospitalised) were lost to follow-up (Figure 2), resulting in dropout rates of 6.5% and 2.5%, respectively. Most deaths in the intervention group occurred in patients who had not yet had any medications deprescribed or had not even undergone a CMR (Supplementary Appendix III), making a causal relationship unlikely.

**Table 1:** Characteristics of patients in the intervention and control group.

Characteristic		Intervention (*n* = 155)	Control (*n* = 163)	*P*-value
**Sociodemographic**				
Sex, female, *n* (%)^a^		89 (57)	85 (52)	.345
Age, years, median (IQR)^b^		82 (78–86)	82 (79–86)	.188
Age, category, *n* (%)	75–79	57 (37)	48 (29)	.370
	80–84	52 (34)	63 (39)	
	>85	46 (30)	52 (32)	
Migration background (yes)^c^, *n* (%)		23 (16)	16 (11)	.187
Complex health problems (ISCOPE >3)^c^, *n* (%)		65 (45)	76 (51)	.315
Low health literacy (SBS-Q < 2)^d^, *n* (%)		42 (29)	42 (28)	.823
**Medication-related**				
Number of medications, median (IQR)^b^		11.0 (10.0–14.0)	12.0 (10.0–14.0)	.472
Number of medications in MDD, median (IQR)^b^		9.0 (7.0–10.0)	9.0 (7.0–10.0)	.547
**Top 10 medication classes (ATC2 level), *n* (%)**				
A02	Drugs for acid-related disorders	147 (95)	151 (93)	
B01	Antithrombotic agents	134 (86)	146 (90)	
C10	Lipid-modifying agents	124 (80)	117 (72)	
C07	Beta-blocking agents	111 (72)	112 (69)	
C09	Agents acting on the renin-angiotensin system	110 (71)	103 (63)	
C03	Diuretics	98 (63)	111 (68)	
A10	Drugs used in diabetes	84 (54)	81 (50)	
A11	Vitamins	68 (44)	50 (31)	
C08	Calcium channel blockers	66 (43)	68 (42)	
C01	Cardiac therapy	62 (40)	67 (41)	

^a^Analysed using Pearson’s chi-square test.

^b^Analysed using the Mann–Whitney U test.

^c^25 missing (14 control, 11 intervention).

^d^26 missing (14 control, 12 intervention).

A total of 304 patients completed the study, providing data for the intention-to-treat analysis. The per-protocol analysis was based on 240 patients, after excluding 64 patients. Patients using fewer than 10 medications at baseline were excluded (*n* = 54; 21 in the intervention group and 33 in the control group). In the intervention group, 10 additional patients were excluded because the planned CMR was not performed.

### Primary outcome

At 6 months, the mean difference in the number of deprescribed medications was 0.79 (95% confidence interval (CI) 0.29–1.28) in favour of the intervention (*P* = .002; Table 2). This reflects a mean of 2.5 medications stopped or reduced per patient in the intervention group (20.7% of baseline medications) compared with 1.7 medications (14.4%) in the control group (Table 2). Per-protocol analysis, restricted to patients who fully met inclusion criteria and, within the intervention group, those who did receive the intervention (*n* = 240), yielded a similar difference of 0.76 medications (*P* = .008). The ICC for the primary outcome was 0.12.

**Table 2:** Medication use and effects on reduced and stopped medications (*n* = 304).

Outcome measure	Intervention		Control	
	Mean (SD)	%	Mean (SD)	%
**Number of medications**				
Baseline (t_0_)	12.1 (2.6)	n/a	11.8 (2.8)	n/a
Follow-up (t_6_)	11.8 (2.6)		11.9 (3.0)	
Δ (t_6_ − t_0_)^a^	−0.30 (1.6)		0.12 (2.0)	
**Medications reduced and/or stopped (% relative to baseline)**				
Reduced & stopped^b^	2.5 (1.7)	20.7	1.7 (1.6)	14.4
Reduced^c^	0.9 (1.0)	7.4	0.4 (0.7)	3.4
Stopped	1.6 (1.3)	13.2	1.3 (1.4)	11.0
**Number of (non)-MDD medications deprescribed (% of total ‘reduced & stopped’)**				
MDD medications	1.3 (1.2)	52.0	0.6 (1.0)	35.3
Non-MDD medications	1.2 (1.3)	48.0	1.1 (1.1)	64.7
**Number of medications started**				
Started	1.3 (1.2)	n/a	1.4 (1.5)	n/a

^a^Between-group difference in change scores (Δ t_6_ − t_0_) analysed using a GLMM with cluster (pharmacy) as random intercept, adjusted for baseline number of medications (β = −0.35, 95% CI −0.78 to 0.08, *P* = .108).

^b^Between-group difference in the number of medications reduced and/or stopped analysed using a GLMM with cluster (pharmacy) as random intercept (β = 0.79, 95% CI 0.29 to 1.28, *P* = .002).

^c^Medication reduction included dose reductions and lower-burden substitutions. Such substitutions occurred in broadly similar proportions in the intervention and control groups (0.15 vs. 0.11 changes per patient; 22/145 vs. 18/159 patients).

In effect modification analyses, a higher baseline medication count was associated with more deprescribing; each additional baseline medication corresponded to an average increase of 0.23 deprescribed medications after 6 months (95% CI 0.17–0.29, *P* < .001). The interaction between baseline medication count and intervention was not statistically significant (β = 0.06, 95% CI −0.07 to 0.18, *P* = .37), indicating that this association was consistent across both groups.

### Secondary outcomes

There was no significant difference in the change in total number of medications between groups (intervention vs control; −0.30 vs +0.12 medications; *P* = .108; Table 2). In the intervention group, an average of 1.6 medications per patient was stopped compared with 1.3 in the control group, while newly initiated medications occurred to a similar extent in both groups (1.3 vs 1.4; Table 2). Dose reduction accounted for almost one medication per patient in the intervention group versus less than half in the control group (0.9 vs 0.4), without affecting the total number of medications. Consequently, the net effect on the total number of medications remained small in both arms. Of the medications deprescribed in the intervention group, approximately half were chronic medications dispensed in the MDD system, compared with one-third in the control group (Table 2). In 127 of the 139 CMRs (91%) in the intervention group, at least one medication was reduced or stopped. Deprescribing was observed across all intervention pharmacies, with a median cluster mean of 2.4 deprescribed medications per patient (IQR 2.0–3.0; range 1.0–3.75).

The number of health problems affecting daily life did not differ between groups at baseline or 6 months (Table 3). The most frequently reported problems were mobility issues (65% control, 71% intervention at baseline), fatigue (61% and 60%) and pain (49% and 56%). At 6 months, these issues remained the most prevalent, with only minor and comparable changes between groups. The change in EQ-VAS scores tended to be higher in the intervention group (mean change +4.1 vs +0.9; *P* = .097; Table 4) but the difference was not statistically significant. EQ-5D utility scores showed no significant change in either group.

**Table 3:** Mean number of health problems with impact on daily life at baseline and follow-up.

Number of health problems with impact	Intervention	Control	*P*-value
Baseline (t_0_), mean (SD)	4.6 (2.9)	4.5 (2.9)	
Follow-up (t_6_), mean (SD)	4.4 (3.2)	4.5 (3.0)	
Δ (t_6_ − t_0_), mean (SD)	−0.14 (2.3)	−0.04 (2.1)	.229^a^

Baseline: 25 missing (11 intervention, 14 control); follow-up: 62 missing (37 intervention, 25 control). Analyses were based on all available data.

^a^ *P*-value for the pairwise contrast of the estimated means at follow-up derived from the GLMM (mean difference −0.47, 95% CI −1.23 to 0.30); model including cluster as a random effect and fixed effects for intervention, time (months), baseline number of health problems, and the time × intervention interaction.

**Table 4:** Effect of the intervention on change in quality of life (EQ-VAS, EQ-5D-5L).

Quality of life measure	Intervention	Control	*P*-value
**EQ-VAS**			
Baseline (t_0_), mean (SD)	63.1 (17.4)	62.9 (15.7)	
Follow-up (t_6_), mean (SD)	67.5 (16.1)	63.6 (15.0)	
Δ (t_6_ − t_0_), mean (SD)	4.1 (19.0)	0.9 (14.0)	.097^a^
**EQ-5D-5L**			
Baseline (t_0_), mean (SD)	0.669 (0.22)	0.698 (0.19)	
Follow-up (t_6_), mean (SD)	0.688 (0.23)	0.677 (0.20)	
Δ (t_6_ − t_0_), mean (SD)	0.014 (0.16)	−0.017 (0.15)	.071^a^

Baseline: 25 missing (11 intervention, 14 control); follow-up: 62 missing (37 intervention, 25 control). Analyses were based on all available data.

^a^ *P*-value for the pairwise contrast of the estimated means at follow-up derived from the GLMM (EQ-VAS: 3.58, 95% CI −0.65 to 7.80; EQ-5D-5L: 0.038, 95% CI −0.003 to 0.079); model including cluster as a random effect and fixed effects for intervention, time (months), baseline value of the outcome, and the intervention × time interaction.

During 139 CMRs in the intervention group, CPs registered 515 DRPs (Supplementary Appendix IV), on average 3.7 per patient. Overtreatment accounted for 34% (*n* = 174), potential adverse effects for 21% (*n* = 108), undertreatment for 13% (*n* = 69) and incorrect dosing for 12% (*n* = 63) of total DRPs. A total of 515 action proposals were made during the CMRs (Table 5), of which 314 (61%) were related to deprescribing. The majority concerned stopping medication (205 proposals, 65% of deprescribing-related actions) or dose reduction (99 proposals, 32%). Implementation rates were 59% and 65%, respectively. Other deprescribing-related action proposals, such as medication substitution, were less common (<3%). In total, 189 deprescribing proposals (60%) were implemented. In 99 CMRs (71%), at least one deprescribing-related action proposal was implemented.

**Table 5:** Number, type and implementation rate of action proposals.

Action proposal	Number *n* (%)	Implemented *n* (%)
Stop medication	205 (40)	120 (59)
Reduce dosage	99 (19)	64 (65)
Substitute medication, e.g. lower potency or reduced treatment complexity	50 (10)	26 (52)
Start medication	46 (9)	28 (61)
Referral to another healthcare provider	22 (4)	21 (96)
Other	22 (4)	16 (73)
Provide information/advice	20 (4)	19 (95)
Increase dosage	19 (4)	16 (84)
Additional monitoring of laboratory values	19 (4)	17 (90)
Discuss use/adherence	13 (3)	13 (100)
**Total**	**515 (100)**	**340 (66)**

Percentages in the ‘Number’ column represent the proportion of total action proposals (*n* = 515).

Percentages in the ‘Implemented’ column represent the proportion of implemented proposals within each category.

Deprescribing was observed across the 10 most frequently used medication classes (ATC level 2) within the MDD system and the corresponding prevalence of deprescribing (Table 6). Among these, deprescribing was most common for drugs for acid-related disorders (A02), drugs used in diabetes (A10) and lipid-modifying agents (C10) in the intervention group. In these classes, between 11% and 28% of users had at least one medication discontinued or reduced, compared with 4%–8% in the control group. Supplementary Appendix V provides a complete overview of all deprescribed MDD medications and shows that deprescribing was largely concentrated in medication classes covered by the deprescribing factsheets [26].

**Table 6:** Top 10 most frequently used medication classes (MDD medication only), ranked by frequency of deprescribing in the intervention group, and corresponding prevalence of deprescribing (*n* = 304).

ATC2	Medication class	Intervention (*n* = 145)		Control (*n* = 159)	
		Total	Deprescribing	Total	Deprescribing
		*n*	*n* (%)	*n*	*n* (%)
A02	Drugs for acid-related disorders	129	37 (29)	145	6 (4)
A10	Drugs used in diabetes	112	16 (14)	103	8 (8)
C10	Lipid-modifying agents	124	14 (11)	125	6 (5)
C03	Diuretics	109	14 (13)	121	8 (7)
C07	Beta-blocking agents	101	13 (13)	108	8 (7)
C01	Cardiac therapy	46	10 (22)	59	4 (7)
A11	Vitamins	55	10 (18)	44	3 (7)
B01	Antithrombotic agents	115	8 (7)	126	9 (7)
C09	Agents acting on the renin–angiotensin system	102	8 (8)	102	12 (12)
C08	Calcium channel blockers	63	3 (5)	68	4 (6)

## Discussion

This study demonstrated that a deprescribing-focused CMR in older patients with hyperpolypharmacy led to an increased number of medications being discontinued or reduced at 6 months, compared with usual care. On average, patients in the intervention group had 2.5 medications deprescribed, compared with 1.7 in the control group. However, the total number of medications in use did not change. No significant changes in health problems or health-related quality of life were observed alongside the increase in deprescribing. CPs identified overtreatment and potential adverse effects as the most common DRPs, and two-thirds of deprescribing recommendations were successfully implemented.

In our trial, patients in the intervention group deprescribed an average of 2.5 medications, of which 1.6 were completely stopped. This aligns with the broader evidence base showing that medication review interventions can slow or reverse the growth in overall medication use. A systematic review of 12 high-quality randomised trials including 1972 patients found that medication review consistently led to a greater decrease or smaller increase in the number of drugs used compared with usual care [32]. In contrast, a previous randomised trial reported a slight increase in medication count after 6 months, our study showed a small, non-significant mean decrease (−0.30 medications in the intervention group vs. +0.12 in controls) [22]. A considerable proportion of the deprescribing actions consisted of dose reductions, which are not reflected in the total medication count. As newly initiated prescriptions occurred to a similar extent in both groups, the small net change in medication count should therefore be interpreted in light of these uncounted dose reductions. This pattern likely reflects both the natural progression of multimorbidity in older adults and the broader objective of the CMR to optimise pharmacotherapy rather than solely deintensify treatment.

The natural progression of multimorbidity may have limited changes within groups over time, thereby reducing the magnitude of any observable effects on health problems or health-related quality of life. In addition, no significant differences between groups were observed for these outcomes. Our study was not designed to detect (small) changes in health problems or quality of life, as these were secondary outcomes mainly aimed at monitoring safety and potential negative outcomes. Importantly, the purpose of deprescribing is not only to solve current problems, but also to limit drug-related risks and decrease unnecessary medication use. In that light, when medication can be deprescribed without an increase in health problems or a decrease in quality of life, this is still a favourable outcome.

Furthermore, our findings on DRPs are broadly consistent with previous national and trial-based studies, which generally report an average of three to five DRPs per patient [22, 32–34]. Variation across studies can largely be explained by contextual factors such as differences in patient populations, the scope and maturity of CMR practice, and the methodological focus of the intervention. For example, studies on national CMR data [33] show similar overall DRP counts but lower proportions of overtreatment, whereas goal-oriented interventions such as the DREAMeR trial [22] identify more DRPs but relatively fewer cases of overtreatment. Against this background, the higher proportion of overtreatment in our study likely reflects the explicit focus of our intervention on deprescribing. This pattern underscores that both the total number of DRPs and the distribution across DRP categories are sensitive to contextual and intervention-specific characteristics.

The deprescribing process involves sequential steps: identifying candidates for dose reduction or discontinuation, implementing deprescribing actions and maintaining persistence over time. Randomised trials have shown variable effectiveness of deprescribing interventions, particularly in primary care, reflecting barriers at each stage. In our trial, 40% of all action proposals involved stopping a medication, whereas in routine Dutch practice fewer than 10% of documented interventions during CMRs concern medication discontinuation [34]. In addition, deprescribing occurred in 71% of CMRs in our study, compared with approximately one-third in routine Dutch practice [34]. This suggests that explicit deprescribing guidance and focus enhance both identification and deprescribing actions.

### Strengths and limitations

A strength of this study is that it was conducted in routine primary care and included a large sample of more than 300 older patients. By targeting patients using MDD systems, deprescribing actions focused on long-term medication use could be objectively monitored through dispensing data and facilitated adherence to tapering schedules by the short dispensing intervals inherent to MDD, which ensured that scheduled dose reductions or discontinuations were implemented and became visible in subsequent dispensing records. Assessing deprescribing after 6 months of follow-up, rather than immediately after the intervention, provided valuable insight into sustained deprescribing. Randomisation at the cluster level minimised contamination between groups, and baseline characteristics were well balanced.

Several limitations should be acknowledged. First, recruitment may have been affected by selection bias, as participating HCPs and patients were likely more motivated or open to deprescribing than average. However, randomisation took place after HCPs participation was confirmed, reducing the likelihood of differential motivation between groups. Second, post-randomisation recruitment by non-blinded clinicians may have introduced recruitment bias. However, all pharmacists were instructed to approach patients without applying a fixed order or reviewing additional patient information to guide invitation. Furthermore, baseline characteristics between groups were well balanced, suggesting no major imbalance resulting from recruitment. Also, findings from our preceding feasibility study indicated that, after applying eligibility and exclusion criteria, pharmacists generally had to approach the large majority of eligible patients in order to achieve the intended inclusion target, limiting the opportunity for selective recruitment [23]. Third, cluster randomisation at the pharmacy level may have introduced between-practice variability. Although clustering was accounted for statistically using mixed models with a random intercept for pharmacy, residual confounding at cluster level cannot be fully excluded. The ICC for the primary outcome (0.12) indicated relevant clustering at pharmacy level, supporting the use of a cluster-adjusted analytical approach. However, the study included a relatively large number of pharmacies with modest cluster sizes, and available pharmacy-level characteristics appeared broadly comparable between groups, which indicates that practice variation related to these characteristic was evenly distributed. Furthermore, although the primary outcome was based on dispensing data, secondary outcomes based on questionnaires had missing data. However, the extent was limited and consistent with expectations for this population, likely reflecting the data collection burden and the frailty of the study population. Finally, although deprescribing guidelines were available to control group participants and deprescribing occurs as part of routine care, they were not actively supported by training. This may have led to some contamination and attenuation of the observed intervention effect, resulting in a more conservative estimate of its true impact. At the same time, findings likely underscores the importance of combining structured training at HCP level with a structured patient-level intervention when implementing such materials in clinical practice.

### Implications for practice and research

Our findings demonstrate that deprescribing can be integrated into CMRs in primary care and effectively stop and reduce medications in an older population on MDD by trained and motivated HCPs. Earlier Dutch research showed that when CMRs include an explicit focus on deprescribing, substantially more actions are implemented and significantly more dose reductions or discontinuations occurred, but this study primarily targeted cardiometabolic medication [25]. This trial shows that providing training and practical tools for HCPs enhances the implementation of deprescribing and increases the proportion of related actions on a range of medications in patients with hyperpolypharmacy. This is supported by the overlap between the medication classes most frequently deprescribed (Supplementary Appendix V) and those addressed in the training materials, suggesting that structured guidance and practical tools are essential for achieving systematic deprescribing across therapeutic domains and should be incorporated into routine CMR practice. Furthermore, patients with a higher baseline medication count experienced more deprescribing overall, suggesting that those with the greatest medication burden may be particularly relevant candidates for deprescribing-focused CMRs.

In this trial, health problems and health-related quality of life remained rather similar over 6 months. A lack of improvement on such outcomes aligns with evidence from other interventions among patients with polypharmacy and multimorbidity, in which measurable improvements in patient-reported outcomes were often difficult to demonstrate over relatively short follow-up periods despite meaningful changes in care processes or medication use [35, 36]. As deprescribing in frail older adults is often gradual and iterative, a single CMR with 6 months of follow-up may not fully capture longer-term effects [35]. In our study, these outcomes were included primarily as indicators of potential negative effects of deprescribing but the trial was not powered to detect differences. Nonetheless, no evidence of short-term deterioration was observed indicative of adverse drug withdrawal events. Future research should evaluate the long-term outcomes of deprescribing within CMRs, including effects on clinical endpoints, healthcare utilisation and costs. In addition, process and implementation evaluations are needed to better understand how deprescribing interventions are delivered, adopted and sustained in routine care, while economic evaluations may help determine whether deprescribing-focused CMRs provide sufficient value relative to their time investment. Studies are also needed to assess the appropriateness of deprescribing decisions in daily practice and to explore strategies for integrating deprescribing into other pharmacist- or physician-led interventions, to support broader and more sustainable implementation in routine care. Additionally, further work could examine how training and experience influence the efficiency of deprescribing-focused CMRs, with the aim of reducing time investment while maintaining effectiveness.

## Conclusion

This pragmatic cluster-randomised trial demonstrates that a deprescribing-focused CMR by trained pharmacists was effective in reducing or stopping medications in older patients with hyperpolypharmacy using MDD systems at 6 months, without a reduction in the total number of medications. No changes in patient-reported health problems or health-related quality of life could be observed over 6 months.

## Supplementary Material

Supplementary_materials_afag209

## Research Data Transparency and Availability

De-identified participant data (including the data dictionary), statistical code and supporting study materials will be made publicly available in a research repository following publication. Details regarding repository access will be provided through the ClinicalTrials.gov registration.
