# Supplementary material for: Deprescribing in older patients with hyperpolypharmacy: a cluster-randomised trial in primary care
Source: Age Ageing. 2026 Jul 19;55(7):afag209. doi: 10.1093/ageing/afag209 (PMC13381036; doi:10.1093/ageing/afag209)
Supplement: Supplementary_materials_afag209 [file supplementary_materials_afag209.zip › aa-26-0859-File004.docx]

| **Characteristic** | **Control**  **(26)** | **Intervention (23)** |
| --- | --- | --- |
| **Pharmacist characteristics** | | |
| Age, mean | 39 | 41 |
| Sex, male | 7 (27%) | 4 (17%) |
| **Pharmacy location, n** | | |
| Village  (<15,000 residents) | 7 | 4 |
| Suburban area  (15,000–100,000 residents) | 10 | 9 |
| Big city  (>100,000 residents) | 9 | 10 |
| **Number of patients in practice, n** | | |
| <8,000 | 3 | 3 |
| 8,000–13,000 | 18 | 14 |
| >13,000 | 5 | 6 |
| **Number of patients using multidose drug dispensing systems, n** | | |
| <100 | 0 | 1 |
| 100–199 | 5 | 2 |
| 200–299 | 11 | 2 |
| 300–399 | 5 | 5 |
| ≥400 | 5 | 13 |
| **Number of pharmacists FTE working in pharmacy, mean** | | |
|  | 2,11 | 2,22 |
| **Included patients per cluster, n (%)** | | |
| <5 patients | 9 (34%) | 10 (43%) |
| 5–10 patients | 15 (58%) | 7 (30%) |
| >10 patients | 2 (8%) | 6 (26%) |

**Appendix I: Characteristics of participating pharmacies and pharmacists by study group**

FTE = full-time equivalent.
