# Supplementary material for: Deprescribing in older patients with hyperpolypharmacy: a cluster-randomised trial in primary care
Source: Age Ageing. 2026 Jul 19;55(7):afag209. doi: 10.1093/ageing/afag209 (PMC13381036; doi:10.1093/ageing/afag209)
Supplement: Supplementary_materials_afag209 [file supplementary_materials_afag209.zip › aa-26-0859-File006.docx]

| **ID** | **Baseline characteristics** | | | | | **Time to death (days from t₀)** | **CMR started?** | **Medication changes resulting from the CMR** | | **Other medication changes** | |
| --- | --- | --- | --- | --- | --- | --- | --- | --- | --- | --- | --- |
|  | **Age** | **Sex** | **EQ-VAS** | **EQ5D** | **ISCOPE** |  |  | **Proposals to reduce dosage** | **Proposals to stop medication** | **Reduced** | **Stopped** |
| **Intervention** | | | | | | | | | | | |
| 1 | 77 | M | 50 | 0.486 | 1 | 56 | yes | Pantoprazole 40 mg → 20 mg | - | - | Acetylcysteine 600 mg: 3×/day |
|  |  |  |  |  |  |  |  |  |  |  | Acetylsalicylic acid 80 mg |
|  |  |  |  |  |  |  |  |  |  |  | Colecalciferol 5600 IU: once weekly |
|  |  |  |  |  |  |  |  |  |  |  | Lisinopril 10 mg |
|  |  |  |  |  |  |  |  |  |  |  | Povidone eye drops 50 mg/ml |
| 2 | 84 | V | 50 | 0.239 | 3 | 67 | yes | - | - | Methotrexate tablet 2.5 mg: 3 tablets once weekly → 2 tablets once weekly | Cetomacrogol cream with vaseline 10% |
|  |  |  |  |  |  |  |  |  |  |  | Clotrimazole cream 10 mg/g |
|  |  |  |  |  |  |  |  |  |  |  | Colecalciferol 5600 IU: once weekly |
| 3 | 78 | V | 45 | 0.249 | 3 | 41 | yes | - | - | - | Colecalciferol 50,000 IU: 2 capsules once every 12 weeks |
|  |  |  |  |  |  |  |  |  |  |  | Metoprolol succinate 25 mg: 3 tablets daily |
| 4 | 81 | V | 60 | 0.813 | 2 | 76 | yes | "Pantoprazole: since used only as prophylaxis with clopidogrel and amitriptyline, 20 mg once daily should be sufficient  → consider lowering dose" | Fentanyl: initially used as emotional trigger in family context; now patient has no pain  → GP to consider stopping | - | - |
|  |  |  |  |  |  |  |  |  | Clopidogrel: started by neurologist in 10/2018, but no (current) indication |  |  |
|  |  |  |  |  |  |  |  |  | Nifedipine: repeat BP measurement and depending on outcome consider discontinuation. |  |  |
| 5 | 86 | M | 85 | 0.781 | 1 | 60 | no | - | - | - | Pravastatin 40 mg |
| 6 | 92 | M | 50 | 0.615 | 4 | 58 | yes | - | - | - | Atorvastatin 40 mg |
|  |  |  |  |  |  |  |  |  |  |  | Vaseline lanette cream |
| 7 | 80 | M | 50 | 0.824 | 2 | direct? | yes | - | - | - | - |
| 8 | 87 | M | 60 | 0.813 | 1 | 54 | no | - | - | - | - |
| 9 | 97 | V | n.v.t. | n.v.t. | n.v.t. | 72 | no | - | - | - | Acenocoumarol 1 mg; schedule |
| **Control** | | | | | | | | | | | |
| 1 | 87 | M | 30 | 0.411 | 4 | 16 | N/A | N/A | N/A | - | - |
| 2 | 90 | V | n.v.t. | n.v.t. | n.v.t. | 69 | N/A | N/A | N/A | - | - |
| 3 | 88 | V | n.v.t. | n.v.t. | n.v.t. | direct? | N/A | N/A | N/A | - | - |

**Appendix III: Deceased patients and follow-up**
