# Supplementary material for: Deprescribing in older patients with hyperpolypharmacy: a cluster-randomised trial in primary care
Source: Age Ageing. 2026 Jul 19;55(7):afag209. doi: 10.1093/ageing/afag209 (PMC13381036; doi:10.1093/ageing/afag209)
Supplement: Supplementary_materials_afag209 [file supplementary_materials_afag209.zip › aa-26-0859-File007.docx]

| **Action proposal (n)** | **Drug related problem (n)** | | | | | | | **Total (n)** |
| --- | --- | --- | --- | --- | --- | --- | --- | --- |
|  | Overtreatment | (potential) side effect | Undertreatment | Wrong dosage | Inconvenience of use | Drug not effective | Contra indication or interaction |  |
| Stop medication | 141 | 37 | 0 | 0 | 6 | 20 | 1 | 205 |
| Reduce dosage | 25 | 29 | 0 | 42 | 1 | 0 | 2 | 99 |
| Replace medication | 2 | 19 | 4 | 1 | 11 | 12 | 1 | 50 |
| Start medication | 0 | 2 | 43 | 0 | 0 | 1 | 0 | 46 |
| Referral to another healthcare provider | 2 | 3 | 10 | 1 | 2 | 4 | 0 | 22 |
| Other | 0 | 1 | 0 | 3 | 18 | 0 | 0 | 22 |
| Provide information/advice | 0 | 9 | 7 | 0 | 3 | 0 | 1 | 20 |
| Increase dosage | 0 | 1 | 2 | 14 | 0 | 2 | 0 | 19 |
| Additional monitoring of laboratory values | 3 | 6 | 3 | 0 | 0 | 2 | 5 | 19 |
| Discuss use/adherence | 1 | 1 | 0 | 2 | 7 | 1 | 1 | 13 |
| **Total (n)** | **174** | **108** | **69** | **63** | **48** | **42** | **11** | **515** |

**Appendix IV: Number and types of drug-related problem and associated action proposals**
