# Supplementary material for: Deprescribing in older patients with hyperpolypharmacy: a cluster-randomised trial in primary care
Source: Age Ageing. 2026 Jul 19;55(7):afag209. doi: 10.1093/ageing/afag209 (PMC13381036; doi:10.1093/ageing/afag209)
Supplement: Supplementary_materials_afag209 [file supplementary_materials_afag209.zip › aa-26-0859-File008.docx]

| **Appendix V: Deprescribed medication (MDD medication only) in relation to deprescribing fact sheets [26]** | | | | | | | |
| --- | --- | --- | --- | --- | --- | --- | --- |
|  |  |  |  |  | |  | |
| **ATC2-code** | **ATC5-code** | **Drug** | **Related to deprescribing protocol**  **(yes/no)** | **Intervention**  **(n=145)** | | **Control**  **(n=159)** | |
|  |  |  |  | **Reduced*** | **Stopped** | **Reduced*** | **Stopped** |
|  |  |  |  | **n** | **n** | **n** | **n** |
| A02 | A02BC01 | Omeprazole | yes | 10 | 2 | 2 | 2 |
|  | A02BC02 | Pantoprazole | yes | 18 | 4 | 1 | 1 |
|  | A02BC05 | Esomeprazole | yes | 2 | 1 | 0 | 0 |
| A03 | A03AA04 | Scopolamine | no | 1 | 0 | 0 | 0 |
| A10 | A10BA02 | Metformin | yes | 7 | 2 | 4 | 1 |
|  | A10BB03 | Gliclazide | yes | 1 | 0 | 0 | 1 |
|  | A10BB09 | Glimepiride | yes | 3 | 1 | 0 | 2 |
|  | A10BK01 | Sitagliptin | yes | 1 | 1 | 0 | 0 |
| A11 | A11CC03 | Ergocalciferol | no | 0 | 1 | 0 | 0 |
|  | A11CC05 | Colecalciferol | yes | 2 | 6 | 0 | 3 |
| A12 | A12AA04 | Calcium carbonate | yes | 0 | 1 | 0 | 0 |
|  | A12AX | Calcium/Vitamin D combinations | yes | 0 | 1 | 0 | 0 |
| B01 | B01AA04 | Phenprocoumon | yes | 0 | 0 | 0 | 1 |
|  | B01AC04 | Clopidogrel | yes | 0 | 4 | 1 | 2 |
|  | B01AC06 | Acetylsalicylic acid | yes | 0 | 0 | 1 | 1 |
|  | B01AC24 | Ticagrelor | yes | 0 | 1 | 0 | 0 |
|  | B01AF01 | Dabigatran etexilate | yes | 1 | 0 | 0 | 1 |
|  | B01AF02 | Rivaroxaban | yes | 2 | 0 | 1 | 1 |
| B03 | B03AA02 | Ferrous sulfate | no | 0 | 2 | 1 | 2 |
|  | B03AA07 | Ferric hydroxide | no | 1 | 0 | 0 | 0 |
|  | B03BB01 | Cyanocobalamin | no | 0 | 0 | 0 | 1 |
| C01 | C01BD01 | Amiodarone | no | 0 | 1 | 0 | 1 |
|  | C01DA14 | Isosorbide mononitrate | no | 8 | 1 | 2 | 1 |
| C02 | C02CA04 | Doxazosin | yes | 0 | 0 | 0 | 1 |
| C03 | C03AA03 | Hydrochlorothiazide | yes | 0 | 3 | 1 | 2 |
|  | C03BA04 | Chlortalidone | yes | 0 | 1 | 0 | 0 |
|  | C03BA11 | Indapamide | yes | 1 | 0 | 0 | 0 |
|  | C03CA01 | Furosemide | yes | 4 | 2 | 0 | 1 |
|  | C03CA02 | Bumetanide | yes | 1 | 1 | 0 | 1 |
|  | C03DA01 | Spironolactone | yes | 1 | 0 | 1 | 2 |
| C07 | C07AA05 | Propranolol | yes | 1 | 0 | 0 | 0 |
|  | C07AA07 | Nadolol | yes | 0 | 0 | 1 | 1 |
|  | C07AB02 | Metoprolol | yes | 7 | 4 | 3 | 1 |
|  | C07AB07 | Bisoprolol | yes | 0 | 1 | 0 | 2 |
| C08 | C08CA01 | Amlodipine | yes | 0 | 2 | 0 | 3 |
|  | C08CA05 | Nifedipine | yes | 0 | 0 | 0 | 1 |
|  | C08CA13 | Lercanidipine | yes | 0 | 1 | 0 | 0 |
| C09 | C09AA02 | Enalapril | yes | 0 | 0 | 2 | 0 |
|  | C09AA03 | Lisinopril | yes | 0 | 3 | 1 | 0 |
|  | C09AA04 | Perindopril | yes | 0 | 1 | 1 | 0 |
|  | C09AA09 | Fosinopril | yes | 1 | 0 | 0 | 0 |
|  | C09CA01 | Losartan | yes | 1 | 0 | 1 | 0 |
|  | C09CA03 | Valsartan | yes | 0 | 0 | 2 | 0 |
|  | C09CA04 | Candesartan | yes | 0 | 1 | 1 | 0 |
|  | C09CA06 | Olmesartan | yes | 0 | 0 | 3 | 0 |
|  | C09DX04 | Sacubitril + Valsartan | yes | 1 | 0 | 1 | 0 |
| C10 | C10AA01 | Simvastatin | yes | 1 | 3 | 2 | 1 |
|  | C10AA03 | Pravastatin | yes | 1 | 2 | 0 | 1 |
|  | C10AA05 | Atorvastatin | yes | 1 | 2 | 1 | 1 |
|  | C10AA07 | Rosuvastatin | yes | 0 | 2 | 0 | 0 |
|  | C10AX09 | Ezetimibe | no | 0 | 2 | 0 | 0 |
| G04 | G04BD08 | Solifenacin | yes | 0 | 2 | 0 | 1 |
|  | G04CA01 | Alfuzosin | yes | 0 | 1 | 0 | 1 |
|  | G04CA02 | Tamsulosin | yes | 0 | 8 | 0 | 0 |
|  | G04CB01 | Finasteride | yes | 0 | 0 | 0 | 1 |
|  | G04CB02 | Dutasteride | yes | 0 | 2 | 0 | 0 |
| H02 | H02AB06 | Prednisone | no | 2 | 0 | 1 | 2 |
| H03 | H03AA01 | Levothyroxine | no | 0 | 0 | 1 | 0 |
| J01 | J01XE01 | Fosfomycin | no | 0 | 2 | 0 | 0 |
| L02 | L02BA01 | Tamoxifen | no | 0 | 1 | 0 | 1 |
| L04 | L04AX03 | Methotrexate | no | 3 | 0 | 1 | 0 |
| M01 | M01AH05 | Etoricoxib | no | 1 | 0 | 0 | 0 |
| M04 | M04AA01 | Allopurinol | no | 0 | 1 | 0 | 2 |
|  | M04AC01 | Colchicine | no | 0 | 2 | 0 | 0 |
| M05 | M05BA04 | Alendronic acid | yes | 2 | 1 | 1 | 2 |
|  | M05BA07 | Ibandronic acid | yes | 1 | 0 | 0 | 0 |
| N02 | N02AA05 | Oxycodone | no | 1 | 0 | 0 | 0 |
|  | N02AJ13 | Oxycodone + naltrexone | no | 0 | 0 | 1 | 0 |
|  | N02AX02 | Tramadol | no | 0 | 0 | 1 | 0 |
|  | N02BF02 | Paracetamol + tramadol | no | 0 | 2 | 0 | 0 |
| N03 | N03AX14 | Pregabalin | no | 1 | 0 | 0 | 0 |
| N04 | N04BC05 | Ropinirole | no | 0 | 1 | 0 | 1 |
| N05 | N05AH04 | Quetiapine | no | 1 | 0 | 0 | 0 |
|  | N05AN01 | Lithium carbonate | no | 0 | 0 | 1 | 0 |
|  | N05BA04 | Oxazepam | no | 1 | 0 | 0 | 0 |
|  | N05BA06 | Lorazepam | no | 1 | 0 | 0 | 0 |
|  | N05CD07 | Zolpidem | no | 0 | 0 | 0 | 1 |
|  | N05CF01 | Zopiclone | no | 0 | 1 | 0 | 0 |
| N06 | N06AA09 | Amitriptyline | no | 2 | 1 | 1 | 0 |
|  | N06AA10 | Nortriptyline | no | 1 | 0 | 0 | 0 |
|  | N06AB05 | Paroxetine | no | 1 | 0 | 0 | 0 |
|  | N06AB06 | Sertraline | no | 0 | 0 | 1 | 0 |
|  | N06AX11 | Mirtazapine | no | 1 | 1 | 0 | 0 |
| N07 | N07AA02 | Nicotine | no | 1 | 0 | 0 | 0 |
|  | N07CA01 | Acetylcysteine | no | 1 | 1 | 0 | 1 |
| R06 | R06AX27 | Desloratadine | no | 0 | 2 | 0 | 0 |
| **Total** | | | | **99** | **89** | **43** | **52** |

^*^Medication reduction included dose reductions and lower-burden substitutions. Such substitutions occurred in broadly similar proportions in the intervention and control groups for MDD-medication (13/145 vs. 9/159 patients).
